# Effect of lifestyle modifications on anthropometric, clinical, and biochemical parameters in adolescent girls with polycystic ovary syndrome: a systematic review and meta-analysis

**DOI:** 10.1186/s12902-020-00552-1

**Published:** 2020-05-19

**Authors:** Somayeh Abdolahian, Fahimeh Ramezani Tehrani, Mina Amiri, Delaram Ghodsi, Razieh Bidhendi Yarandi, Mahdi Jafari, Hamid Alavi Majd, Fatemeh Nahidi

**Affiliations:** 1 Student Research Committee, Department of Midwifery and Reproductive Health, School of Nursing and Midwifery, Shahid Beheshti University of Medical Sciences, Tehran, Iran; 2 Reproductive Endocrinology Research Center, Research Institute for Endocrine Sciences, Shahid Beheshti University of Medical Sciences, Tehran, Iran; 3 Department of Nutrition Research, Faculty of Nutrition and Food Technology, National Nutrition and Food Technology Research Institute, Shahid Beheshti University of Medical Sciences, Tehran, Iran; 4 Department of Clinical Psychology, School of Medicine, Shahid Beheshti University of Medical Sciences, Tehran, Iran; 5 Department of Biostatics, School of Paramedicine, Shahid Beheshti University of Medical Sciences, Tehran, Iran; 6 Department of Midwifery and Reproductive Health Research Center, Department of Midwifery and Reproductive Health, School of Nursing and midwifery, Shahid Beheshti University of Medical Sciences, Cross of Vali-Asr and Neiaiesh Highway, Opposite to Rajaee Heart Hospital, Tehran, Postal Code: 1996835119 Iran

**Keywords:** Lifestyle, Polycystic ovarian syndrome, Exercise, Diet, Metabolic, Hormonal, Prospero systematic review registration number: CRD42020150812.

## Abstract

**Background:**

Polycystic ovary syndrome (PCOS) is the most common endocrine disorder in women of childbearing age. This study aimed to compare the effects of lifestyle interventions on anthropometric, clinical, and biochemical parameters in adolescent girls with PCOS.

**Methods:**

PubMed, Scopus, and Web of Science was systematically searched to retrieve studies investigating the effects of lifestyle modifications in adolescent girls with PCOS, which were published up to December 2019. The primary outcome was Body Mass Index (BMI) and secondary outcomes were all manifestations of PCOS, including clinical, metabolic, and hormonal parameters. Random effect meta-analysis was applied for significant results. Publication bias was assessed using the Egger test.

**Results:**

This study showed significant improvements in luteinizing hormone (LH) (Pooled SMD = − 0.1.23; 95% CI, − 2.44 to − 0.03), and Free Androgen Index (FAI) levels (Pooled SMD = − 0.78 95% CI, − 0.1.42 to − 0.13) in adolescent girls receiving lifestyle intervention compared to baseline. This study also revealed that diet modifications alone were associated with a significant decrease in Body Mass Index (BMI) (Pooled SMD = − 0.45; 95% CI, − 0.76 to − 0.13), and FG score (Pooled SMD = − 0.81; 95% CI, − 1.33 to − 0.28). Exercise interventions were associated with significant changes in the menstrual cycles (Pooled SMD = 1.16; 95% CI, 0.72 to 1.61), Ferriman-Gallwey (FG) score (Pooled SMD = − 0.57; 95% CI, − 0.99 to − 0.15), LH (Pooled SMD = − 056; 95% CI, − 0.98 to − 0.14), Anti-Müllerian Hormone (AMH) (Pooled SMD = − 0.81; 95% CI, − 0.1.24 to − 0.38), and Triglyceride (TG) levels (Pooled SMD = − 0.32; 95% CI, − 0.62 to − 0.02).

**Conclusion:**

This meta-analysis concluded lifestyle interventions, such as diet and exercise, can improve some clinical, metabolic, and hormonal parameters in adolescent girls with PCOS.

## Background

Polycystic ovary syndrome (PCOS) is the most common endocrine disorder in women of childbearing age [1]. Although the precise prevalence of PCOS in adolescent girls is still unknown, a recent meta-analysis conducted on this age group estimated it to be 3.39 and 11.4% based on the National Institute of Health (NIH) and Rotterdam criteria, respectively [2]. This syndrome is characterized by ovulation irregularities, clinical with or without biochemical hyperandrogenism, and polycystic ovaries [2]. In addition to the clinical and hormonal features, this disease is often associated with an increased risk of metabolic disturbances, such as obesity, dyslipidemia, insulin resistance, and type 2 diabetes mellitus, which predispose patients for cardiovascular diseases [3]. Insulin resistance is prevalent in both lean and obese women with PCOS and is seen in adolescents with hyperandrogenism and in prepubertal girls with early adrenarche [4]. Although PCOS begins at puberty, the source of ovarian androgen production disorder at puberty originates in childhood or even during fetal development [5]. The pathological features in adolescence are still debated since most diagnostic criteria, such as menstrual irregularity, hirsutism, acne, and polycystic ovary morphology (PCOM) are common in normal adolescent females and considered physiologic changes of puberty [6].

Several treatment options are available for managing adolescent girls with PCOS [7]. Recommended treatments, especially in this age group, should be safe, acceptable, and tolerable [8]. However, there are no sufficient data available regarding the safety of pharmacological treatments, and especially related to long term usage in young women with PCOS [9]. Additionally, none of these drugs have been approved so far by the US Food and Drug Administration (FDA) to use in adolescents with PCOS [10].

Lifestyle modification (LSM) is considered as an effective and safe option and the first line of treatment in adolescent girls [11]. Lifestyle interventions, which mainly include dietary change and physical activity, can reduce the prevalence of obesity and hormonal disorders in adolescents [12]. This non-invasive intervention, especially in adolescents, causes 5 to 10% weight loss in obese girls with PCOS [13], and can decrease androgen levels and menstrual cycle irregularities [14]. A systematic review has shown that LSM can improve clinical, hormonal, and metabolic parameters of PCOS in young patients [6, 15].

Despite several existing studies on PCOS in adolescents, a limited number of studies have evaluated the efficacy of treatments of PCOS, in particular LSM in this age group, and have reported conflicting results [10, 16–19]. In addition, to the best of our knowledge, there has been no meta-analysis assessing the effects of LSM on PCOS symptoms in adolescent girls; hence, this study aimed to evaluate the effects of LSM on anthropometric, clinical, and biochemical parameters in adolescent girls with PCOS.

## Methods

The current meta-analysis was designed based on the guidelines for the preferred reporting items for systematic reviews and meta-analyses (PRISMA) [20]. The PICOT question of the study was: What are the effects of lifestyle modifications on the anthropometric, clinical, and biochemical parameters in adolescent girls with PCOS after 3–12 months of intervention. The study protocol was registered in PROSPERO with CRD42020150812 number.

### Search strategy

PubMed, Web of Science, Scopus and Cochrane Library was searched to retrieve studies that were published up to December of 2019. The studies were limited to those that focused on investigating lifestyle modifications in adolescent girls with PCOS. Two reviewers (S.A., M.A.) performed searches separately. Keywords used in the search included:

(“life style” OR “lifestyle” OR “Life Change Events” OR “weight loss” OR “modification” OR “diet” OR “nutrition” OR “nutritional status” OR “food” OR “energy intake” OR “calorie” OR “exercise” OR “physical activity” OR “fitness” OR “behavior” OR “psychiatry” OR “psychology” OR “stress” OR “anxiety” OR “alcohols” OR “drinking” OR “alcohol drinking” OR “smoking”) AND (“polycystic ovarian syndrome” OR “polycystic Ovary Syndrome” OR “PCOS” OR “Stein Leventhal Syndrome”) AND (“adolescent” OR “adolescence” OR “teens” OR “teen” OR “teenagers” OR teenager” OR “youth” OR “youths” OR “child”).

The search was limited to human studies and English language publications. Search strategies were almost the same for all databases, which were conducted based on the ‘all fields’ in the PubMed and ‘titles, abstracts, and keywords’ in other databases. We also appraised the reference lists of all included studies for any additional publications that could be used in this review.

### Eligibility criteria

All clinical trials published in the English language, without any time limitation that investigated lifestyle interventions/modifications in a study population of adolescent girls with PCOS were included in the study. Non-clinical trial studies that evaluated an adult population, did not mention diagnostic PCOS criteria, used drug interventions in combination with lifestyle interventions, or those with unreliable and incomplete results were omitted from the study. The intervention of interest was lifestyle modification/interventions, including nutrition interventions (diet therapy or nutrition intervention including nutrition education or nutrition/dietary consulting at schools, or medical centers), physical activity interventions (exercise or fitness or yoga) and behavior interventions.

### Outcome measure

The primary outcome was Body Mass Index (BMI) and the secondary outcomes were all manifestations of PCOS, including clinical [FG score, menstrual cycles, BMI and Blood Pressure (BP)], metabolic parameters [Fasting Blood Sugar (FBS), Fasting Blood Insulin (FBI), Homeostatic Model Assessment - Insulin Resistance (HOMA-IR), Triglyceride (TG), Low-Density Lipoprotein (LDL), High-density Lipoprotein (HDL)], and hormonal parameters [Sex Hormone Binding Globulin (SHBG), Follicle-Stimulating Hormone (FSH), LH, Total Testosterone (TT), and Bioavailable Testosterone (BT), AFI, AMH].

### Study selection

All relevant clinical trials investigating the effect of lifestyle modification/interventions on PCOS manifestations in adolescent girls with PCOS were included. At least one of the following outcomes had to be reported: hirsutism, menstrual cycles, and androgenic, metabolic parameters such as FBS, FBI, HOMA-IR, TG, TC, LDL, HDL, anthropometric parameters, and blood pressure. After initial screening by one reviewer (SA), the potentially eligible studies were entered into the software Endnote. The first selection was performed based on their titles, followed by a second selection performed by one reviewer who deleted duplicates and reviewed the abstracts of all remaining records. If there was any difference of opinions in the selection of abstracts, it was resolved by consensus or by another reviewer. Full-text papers of all selected abstracts were obtained for reviewing and data processing.

### Data extraction

For minimizing errors, two reviewers (S.A. and M.A.), in close consultation with senior reviewers, extracted data from full-text articles and double-checked all data extracted to minimize errors. The following data were extracted (if available): author, publication year, country, methodology, including criteria for sample size, study design, time of intervention, duration of intervention, and main outcomes. Any disagreement was settled by discussion or, if required, consultation with a third person and via the consensus strategy. We documented the selection process in sufficient detail to complete a PRISMA flow diagram.

### Quality assessment

The two reviewers (S.A. and M.A.) who were blinded to the study author, institution, journal name, volume, and page, assessed the quality of each study separately. Any dispute was resolved and adjusted by the senior reviewer (F.R.T.). The most important items of the Modified Consolidated Standards of Reporting Trials (CONSORT) checklist were used to assess the quality of RCTs [21]. In this respect, the quality of RCTs was individually evaluated using predefined criteria. High-quality studies were defined as total score > 60%, fair quality (40–60% score), and poor quality (< 40% score) (Table 1 in Additional file 1).

### Risk of bias assessment

The two authors (S.A. and M.A.) independently assessed the risk of bias in each study included using the criteria outlined in the Cochrane Handbook for Systematic Reviews of Interventions [22]. Seven domains related to the risk of bias were assessed in each RCT: (1) random sequence generation, (2) allocation concealment, (3) blinding of outcome assessment, (4) comparison with control group, (5) incomplete outcome data, (6) selective outcome reporting, and (7) comparison with baseline. Review authors’ conclusions were considered as ‘low risk’, ‘high risk’ and ‘unclear risk’ of bias (Table 2 in Additional file 1).

### Statistical analysis

This meta-analysis was conducted to obtain the pooled standardized mean difference of clinical features (BMI, menstrual cycle, and FG), blood pressure (SBP and DBP), and lipid profiles (TG, HDL, and LDL), glucose metabolism (FBS, FBI, and HOMA-IR), hormone profiles (FSH, LH, AMH, SHBG, FT, BT, TT, and AFI).

Means and SDs of data at baseline and after treatment were collected. For effect measures, the mean difference (MD) and related 95% confidence intervals (CIs) were calculated based on means of pre-treatment and those at end of treatment levels.

Forest plots summarize the mean difference (CI 95%) by using variance between studies and the random effect model. I-squared statistics were estimated as the measures of heterogeneity. Random effect meta-analysis was applied for significant results (I2 greater than 50% or the Chi-squared test), otherwise the fixed-effect model was applied. Publication bias was assessed using the Egger test [23]. Meta-regression analyses were also conducted to adjust lifestyle intervention types and follow-up time as a confounding variable.

## Results

### Search results, study selection, study characteristics, and quality assessment

**Fig. 1:**
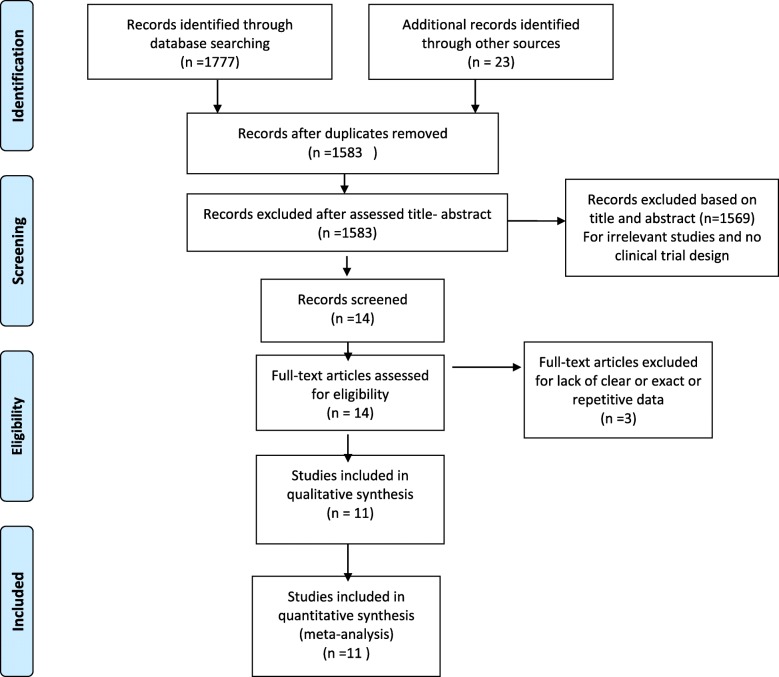
PRISMA flow diagram of study selection

A flow chart of the literature search and its results are shown in Fig. 1. We screened a total of 1777 records from the following databases: Web of Science (366), PubMed (188), and Scopus (1224). Finally, eleven studies were included in the meta-analysis; of these, five studies were classified as having high quality, four studies as fair, and two as low quality (Table 1 in the supplementary file). Therefore, most studies included in the meta-analysis had moderate quality. The study population consisted of 412 adolescent girls with PCOS in 16 interventions and 3 control groups. Five studies with lifestyle interventions (the combination of dietary, physical activity, and behavior habits intervention) [24–28], four studies with dietary interventions [29–32], and two studies with exercise interventions were entered in this study [33, 34]. The characteristics of these studies are shown in Table 1.

**Table 1:** Characteristics of studies included

Author (year) setting	Study design	Sample size	Groups (mean age and bmi)	PCOS diagnosis criteria	Life style modification	Follow up duration	outcome	Main result
Wong et al., 2016 USA	RCT	19	Group 1: patient with diet intervention and Weight loss *n* = 9 Mean age:16.3 ± 2.2 Mean BMI baseline: 32.80 ± 3.20 Mean BMI after:30.9 ± 3.70 Group 2: patient with diet intervention and non-Weight loss *n* = 10 Mean age:15.4 ± 1.3 Mean BMI baseline:36.50 ± 4.30 Mean BMI after:36.10 ± 4.70	Androgen Excess Society	Diet LGL (45% carbohydrate, 35% fat, 20% protein) or LF (55% carbohydrate, 25% fat, 20% protein) diet	6 months	BMI, BP,FBS FBI,SHBG,TG HDL, LDL,TT FT, BT	↓BMI
Lass et al., 2011 German	NRS	59	Group 1: patient with lifestyle intervention and Weight loss *n* = 26 Mean age:14.9 ± 0.8 Mean BMI baseline:32.20 ± 3.70 Mean BMI after:28.30 ± 3.40 Group 2: patient with lifestyle intervention and non-Weight loss *n* = 33 Mean age:15.1 ± 0.7 Mean BMI baseline:33.90 ± 6.8 Mean BMI after:34.60 ± 6.90	National Institutes of Health	Diet 30% fat, 15% proteins, and 55% carbohydrates including 5% sugar Exercise dancing, ball games, jogging, trampoline jumping Behavior	12 months	BMI, BP,FBS FBI, SHBG HOMA, TG HDL, LDL, FSH LH, FT, AFI	↓BMI, ↓TG, ↓HOMA, ↓testosterone, ↓FAI, ↓LH, ↓systole and ↓diastole blood pressure
Reinehr et al., 2017 German	NRS	20	Group 1: patient with lifestyle intervention and Weight loss *n* = 10 Mean age:: 14.9 ± 1.4 Mean BMI baseline:32.20 ± 4.10 Mean BMI after: 27.40 ± 2.5 Group 2: patient with lifestyle intervention and non-Weight loss *n* = 10 Mean age:15 ± 1.2 Mean BMI baseline:31.40 ± 4.90 Mean BMI after:33.30 ± 6.6	Endocrine Society proposed	Diet 30% fat, 15% proteins, and 55% carbohydrates including 5% sugar Exercise dancing, ball games, jogging, trampoline jumping Behavior	12 months	BMI, SHBG HOMA, FSH LH, FT, AFI AMH	↓BMI, ↓LH, ↑SHBG, ↓FAI, ↓HOMA, ↓AMH
Hoeger et al., 2008 USA	RCT	18	Group 1: patient with lifestyle intervention *n* = 8 Mean age: 15.4 ± 1.2 Mean BMI baseline:36 ± 6.20 Mean BMI after: 34.90 ± 7 Group 2: patient with no intervention (control) *n* = 10 Mean age: 15.4 ± 1.7 Mean BMI baseline:34.90 ± 6.7 Mean BMI after: 34.90 ± 6.70	Rotterdam criterion	Diet Hypo caroric diet with 500 kcal/d deficit Exercise:30 min/d of moderate to intense activity	6 months	BMI, BP,FBS FBI, TG, SHBG HDL, LDL, TT AFI, FG	↓FAI, ↑SHBG, ↓Diastolic blood pressure
Ornestein et al., 2011 Chicago	RCT	16	Group 1: patient with diet intervention *n* = 16 Mean age: 15.8 ± 2.2 Mean BMI baseline: 35.70 ± 6 Mean BMI after: 32.90 ± 5.80	Rotterdam criterion	Diet:Low fat less than 40 g per day of fat, with five servings of starch per day and an ad libitum intake of fat-free dairy foods, fruits, vegetables low carbohydrate: 40 g carbohydrate daily by adding low glycemic index foods, such as nuts, fruits, And whole grains.	3 months	BMI	↓BMI
Ladson et al., 2011 USA	RCT	11	Group 1: patient with lifestyle intervention *n* = 11 Mean age: 15.4 ± 1.2 Mean BMI baseline: no reported Mean BMI after: no reporte	National Institutes of Health	Diet: 55% carbohydrate, 30% fat, and 15% protein. Exercise: 35–45 stair stepper, stationary bike, elliptical machine, treadmill, or dancing to music	6 months	BP, FBS, FBI HOMA, TG SHBG, HDL LDL, FSH LH, BT,AFI	No significant difference in all of measurement outcome
Nidhi et al., India 2012	RCT	71	Group 1: patient with yoga intervention *n* = 42 Mean age:16.22 ± 1.13 Mean BMI baseline:20.22 ± 1.65 Mean BMI after:20.11 ± 1.70 Group 2: patient with exercise intervention *n* = 43 Mean age:16.22 ± 0.93 Mean BMI baseline:21.28 ± 3.05 Mean BMI after: 21.59 ± 2.78	Rotterdam criterion	Exercise: A yoga suryanamaskara, asanas, pranayama, and meditation	3 months	BMI, FBS,FBI HOMA, TG HDL, LDL	↓FBS, ↓HOMA, ↓TG, ↓LDL
Nidhi et al., India 2013	RCT	72	Group 1: patient with lifestyle intervention *n* = 45 Mean age: 16.22 ± 1.13 Mean BMI baseline:20.39 ± 2.60 Mean BMI after:20.41 ± 2 Group 2: patient with no intervention (control) *n* = 45 Mean age:16.22 ± 0.93 Mean BMI baseline:21.39 ± 3.20 Mean BMI after:21.70 ± 2.88	Rotterdam criterion	Exercise: A yoga suryanamaskara, asanas, pranayama, and meditation	3 months	BMI, FSH, LH TT, AMH, FG menstrual period	↓TT, ↓LH, ↓AMH, ↓FG, menstural period improve
Carolo et al., 2017 Brazil	NRS	18	Group 1: patient with lifestyle intervention and Weight loss n = 9 Mean age:16.56 ± 1.33 Mean BMI baseline:88.10 ± 13.3 Mean BMI after:81.40 ± 11.20 Group 2: patient with diet intervention and non-Weight loss *n* = 9 Mean age:16 ± 1.66 Mean BMI baseline:78.70 ± 17.30 Mean BMI after:84.8 ± 20.7	Endocrine Society	Diet 55–75% of carbohydrate, 10–15% of Protein and 15–30% of total fat.	6 months	BMI	Dietary interventions were not beneficial for BMI improve.
Rofey et al., 2009 USA	NRS	12	Group 1: patient with lifestyle intervention *n* = 12 Mean age: 15.8 Mean BMI baseline:39 ± 9 Mean BMI after:35 ± 6	National Institutes of Health	Behavior: cognitive–behavioral therapy (CBT) Exercise: (yoga instructor, local swim coach, climbing wall supervisor)	3 months	BMI	↓ BMI.
Marzouk et al., 2015 Egypt	RCT	60	Group 1: patient with diet intervention *n* = 30 Mean age:19.3 ± 1.3 Mean BMI baseline:36.4 ± 4.7 Mean BMI after:33.20 ± 3.8 Group 2: patient with no intervention (control) *n* = 30 Mean age:20.1 ± 1.8 Mean BMI baseline:35.8 ± 4.8 Mean BMI after:35.7 ± 4.67	Rotterdam criterion	Diet 15–20% of protein, 30% of fat, 50–55% of carbohydrates	6 months	BMI, FG menstrual period	↓ BMI, ↓FG, menstrual period improve.

*PCOS* polycystic ovarian syndrome; *BMI* body mass index; *FSH* Follicle-stimulating hormone; *LH* Luteinizing Hormone; *FBS* fasting blood sugar; *HOMA* Homeostatic model assessment; *FBI* Fasting blood index; *TG* Triglyceride; *LDL* Low-density lipoprotein; *HDL* High-density lipoprotein; *SHBG* Sex Hormone Binding Globulin; *TT* total testosterone; *FT* free testosterone; *BT* bioavailable testosterone; *FAI* free androgen index; *AMH* Anti-Müllerian hormone; *BP* Blood pressure; *FG* Ferriman-Gallwey; *LF* Low Fat; *LGL* Low Glycemic Load

Three studies were excluded because of having incomplete outcome data [35], repetitive data [36], and one being a longitudinal design study [37].

The results for risk of bias are shown in Table 2 in the supplementary file. For random sequence generation, seven trials (7 out of 11, 63%) were deemed to be at low risk [26, 29–31, 33, 34, 38], and for allocation concealment, seven trials were at unclear risk (7 out of 11, 63%) [25, 27, 29, 31, 33, 34, 38]. In three trials (3 out of 11, 27%), the participants or providers, or both, were blinded to treatment allocation [24, 33, 34]. Eight trials (8 out of 11, 72%) reported attrition bias [25–27]. All of the included studies were deemed to be at low risk for comparing before-after intervention results and for selecting outcome reports. The intervention and control groups were demonstrably comparable in six trials (6 out of 11, 55%) [30, 33, 34, 38, 39] [26, 27]. Some biases were more probable such as blinding of outcome assessment and allocation concealment.

### Meta-analysis of outcomes

A summary of the key findings reported in each of the outcomes assessed in this meta-analysis is presented in Table 2. Pooled SMD (95% CI) in treatment groups is shown in these tables.

**Table 2:** Results of meta-analysisi and Meta-regression

Outcomes	N	^a^I^2^%	^c^Publication bias	Pooled SMD (95%CI)			^b^Meta-regression coefficient (*P*-Value)
				SMD	LCI	UCI	
BMI							
*Intervention: lifestyle*	4	69.9	0.088	−0.442	− 0.986	0.102	− 0.47 (0.119)
*Intervention: Exercise*	2	0.0	0.125	0.017	−0.226	0.260	−0.038 (0.896)
*Intervention: Diet*	4	0.0	0.147	−0.450	−0.760	− 0.139	− 0.47 (0.128)
*Control*	3	0.0	0.568	0.057	−0.242	0.356	Reference
FG score	1						
*Intervention: lifestyle*		–	–	−0.509	−1.360	0.341	−0.57 (0.344)
*Intervention: Exercise*	1	–	–	−0.575	− 0.996	−0.153	− 0.63 (0.139)
*Intervention: Diet*	1	–	–	− 0.807	− 1.334	−0.280	− 0.86 (0.108)
*Control*	3	–	–	0.056	− 0.243	0.355	Reference
SBP							
*Intervention: lifestyle*	2	72.2	0.602	− 0.070	−0.743	0.602	− 0.335 (0.651)
*Intervention: Exercise*	0	–	–	–	–	–	–
*Intervention: Diet*	1	0	–	0.267	− 0.372	0.907	0 .014 (0.986)
*Control*	1	–	–	0.252	− 0.588	1.091	Reference
DBP							
*Intervention: lifestyle*	2	68.8	0.117	−0.216	− 0.855	0.422	0.135 (0.861)
*Intervention: Exercise*	0	–	–	–	–	–	–
*Intervention: Diet*	1	0	0.317	−0.451	− 1.093	0.191	−0.081 (0.905)
*Control*	1	–	–	− 0.349	− 1.192	0.494	Reference
Menstrual cycle							
*Intervention: lifestyle*	0	–	–	–	–	–	NA
*Intervention: Exercise*	1	–	–	1.165	0.718	1.613	NA
*Intervention: Diet*	1	–	–	0.467	− 0.046	0.980	NA
*Control*	2	53	0.317	0.344	− 0.138	0.826	NA
FBS							
*Intervention: lifestyle*	2	0	–	0.113	−0.219	0.445	0.246 (0.746)
*Intervention: Exercise*	1	94	0.317	−0.530	− 1.808	0.748	− 0.374 (0.636)
*Intervention: Diet*	1	–	–	− 0.092	− 0.729	0.545	0.052 (0.950)
*Control*	1	–	–	− 0.148	− 0.985	0.689	Reference
FBI							
*Intervention: lifestyle*	2	78.3	0.117	−0.066	−0.824	0.693	−0.192 (0.814)
*Intervention: Exercise*	1	85.1	0.317	−0.126	−0.914	0.661	− 0.444 (0.779)
*Intervention: Diet*	1	0	–	− 0.108	−0.749	0.533	−0.234 (0.758)
*Control*	1	–	–	0.126	− 0.711	0.962	Reference
HOMA-IR							
*Intervention: lifestyle*	2	0	0.254	0.000	− 0.312	0.312	–
*Intervention: Exercise*	1	0	0.317	0.000	−0.301	0.301	–
*Intervention: Diet*	0	–	–	–	–	–	–
*Control*	0	–	–	–	–	–	–
TG							
*Intervention: lifestyle*	2	64.1	0.317	−0.097	− 0.705	0.511	− 0.095 (0.235)
*Intervention: Exercise*	1	0	0.317	−0.321	− 0.624	−0.018	− 0.180 (0.584)
*Intervention: Diet*	1	0	0.117	−0.097	−0.705	0.511	−0.041 (0.784)
*Control*	1	–	–	−0.236	−1.075	0.603	Reference
SHBG							
*Intervention: lifestyle*	3	70.1	0.421	0.593	−0.004	1.191	0.341 (0.623)
*Intervention: Exercise*	0	–	–	–	–	–	–
*Intervention: Diet*	1	0	0.117	0.007	− 0.630	0.644	− 0.224 (0.784)
*Control*	1	–	–	0.235	− 0.604	1.074	Reference
HDL							
*Intervention: lifestyle*	2	35.5	0.217	0.317	− 0.115	0.749	−0.075 (0.879)
*Intervention: Exercise*	1	0	0.117	0.089	−0.212	0.389	−0.311 (0.532)
*Intervention: Diet*	1	0	0.117	0.056	−0.582	0.694	−0.343 (0.560)
*Control*	1	–	–	0.400	− 0.445	1.245	Reference
LDL							
*Intervention: lifestyle*	2	0	0.317	−0.059	− 0.390	0.273	0.063 (0.898)
*Intervention: Exercise*	1	54.1%	0.117	−0.431	− 0.882	0.021	−0.301 (0.542)
*Intervention: Diet*	1	0	0.117	−0.455	− 1.100	0.190	−0.333 (0.571)
*Control*	1	–	–	−0.122	− 0.958	0.715	Reference
FSH							
*Intervention: lifestyle*	2	28.2	0.254	0.049	−0.337	0.435	0.202 (0.635)
*Intervention: Exercise*	0	0	–	− 0.207	−0.621	0.207	−0.063 (0.900)
*Intervention: Diet*	0	–	–	–	–	–	–
*Control*	1	–	–	− 0.144	−0.558	0.270	Reference
LH							
*Intervention: lifestyle*	2	28.2	0.254	−1.235	−2.440	− 0.030	−2.115 (0.564)
*Intervention: Exercise*	0	0	–	− 0.561	−0.982	− 0.140	−0.982 (0.894)
*Intervention: Diet*	0	–	–	–	–	–	–
*Control*	1	–	–	0.420	0.003	0.838	Reference
TT							
*Intervention: lifestyle*	1	–	–	0.140	−0.697	0.977	−0.0476 (0.952)
*Intervention: Exercise*	1	–	–	−0.288	− 0.703	0.128	− 0.476 (0.236)
*Intervention: Diet*	1	0	0.117	−0.050	− 0.686	0.586	−0.238 (0.592)
*Control*	1	0	0.117	0.188	−0.183	0.559	Reference
FT	2	80.2					
*Intervention: lifestyle*			0.542	−0.486	− 1.277	0.306	–
*Intervention: Exercise*	0	–	–	–	–	–	–
*Intervention: Diet*	1	81	0.117	−1.042	− 2.737	0.653	–
*Control*	0	–	–	–	–	–	Reference
BT *Intervention: lifestyle*	0	–	–	–	–	–	–
*Intervention: Exercise*	0	–	–	–	–	–	–
*Intervention: Diet*	1	0	–	−0.073	−0.710	0.564	–
*Control*	0	–	–	–	–	–	Reference
AFI							
*Intervention: lifestyle*	3	73.8	0.452	−0.778	− 1.425	−0.131	− 0.918 (0.400)
*Intervention: Exercise*	0	–	–	–	–	–	–
*Intervention: Diet*	0	–	–	–	–	–	–
*Control*	1	0	–	0.119	−0.718	0.955	Reference
AMH							
*Intervention: lifestyle*	1	87.2	0.117	−1.230	−3.259	0.798	−1.084 (0.654)
*Intervention: Exercise*	1	–	–	−0.809	− 1.239	−0.378	−0.622 (0.541)
*Intervention: Diet*	0	–	–	–	–	–	–
*Control*	1	–	–	− 0.187	−0.601	0.227	Reference

^a^Heterogeneity Index: Value upper 50% needs Random effect method of estimation

^b^Meta-regression coefficient showed difference of intervention vs. control

^c^Egger test of publication bias which test small-study effects

### Body mass index

Ten studies reported the effect of lifestyle on BMI, four studies evaluated diet [29–32], two studies evaluated exercise [33, 34] and four studies used a combination of these interventions [25–28].

**Fig. 2:**
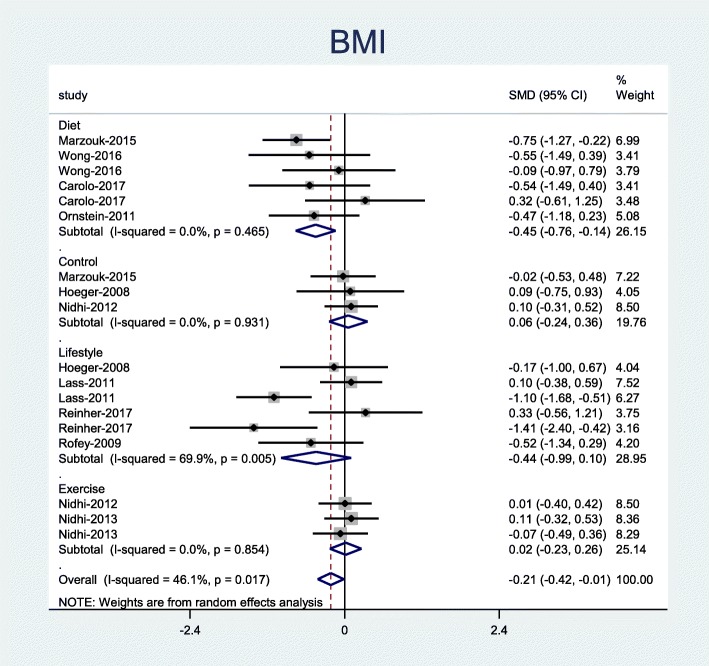
Forest plot of pooled mean difference standardized of BMI

Our results showed that only diet intervention was associated with a significant decrease in BMI (Pooled SMD = − 0.45; 95% CI, − 0.76 to − 0.14) (Fig. 2).

### Clinical parameters

In general, six studies reported the effect of LSM on clinical parameters including menstrual cycles, FG, and systolic and diastolic blood pressure; two of these studies assessed the effect of diet [29, 30], one study evaluated exercise [33], and three studies assessed the combination of diet, exercise, and behavior [24–26].

**Fig. 3:**
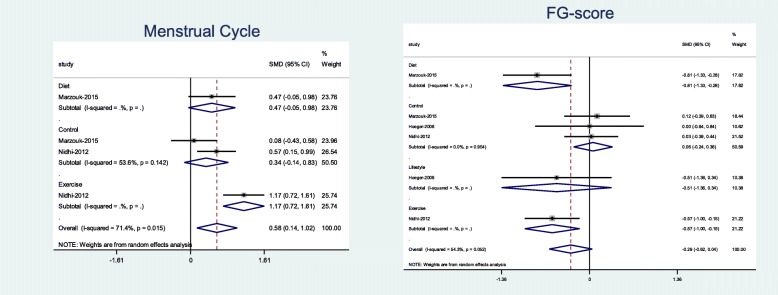
Forest plot of pooled mean difference standardized of menstrual cycle and FG score

Results showed that exercise intervention significantly improved menstrual cycle regularities (Pooled SMD = 1.16; 95% CI, − 0.72 to 1.61) and the FG score (Pooled SMD = − 0.57; 95% CI, − 0.99 to − 0.15). In addition, diet therapy was associated with a significant decrease in FG scores (Pooled SMD = − 0.81; 95% CI, − 1.33 to − 0.28) (Fig. 3).

Lifestyle intervention had no significant effect on systolic or and diastolic blood pressure (Fig. 1 in Additional file 1).

### Metabolic parameters

In general, six studies reported on the effect of one lifestyle intervention type on metabolic parameters including TG, HDL, LDL, FBS, FBI, and HOMA-IR. Another study evaluated the effect of diet [29], one study evaluated the effect of exercise [34], and four studies evaluated the effect of a combination of diet, exercise, and behavior modification [24–27].

**Fig. 4:**
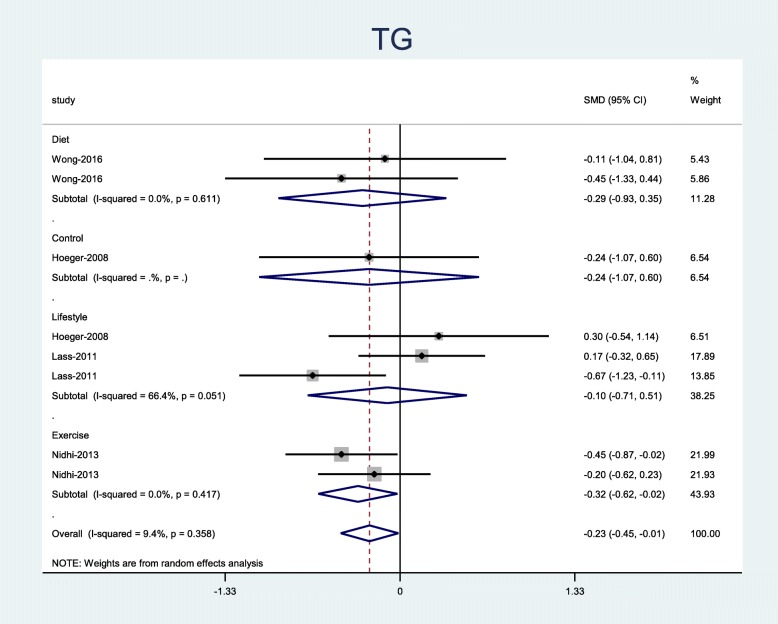
Forest plot of pooled mean difference standardized OF TG

Results revealed that exercise intervention significantly decreased the TG level (Pooled SMD = − 0.32; 95% CI, − 0.62 to − 0.02) (Fig. 4). This study showed that lifestyle interventions (diet, exercise, and behavior) had no significant effects on HDL, LDL, FBS, FBI, and HOMA-IR (Figs. 2, 3, 4 in Additional file 1).

### Hormonal parameters

In general, six studies reported the effect of LSM on metabolic parameters, including FSH, LH, SHBG, TT, FT, BT, FAI, and AMH. One of these studies evaluated the effect of diet [29], one study evaluated the effect of exercise [33], four studies evaluated effects of the combination of these interventions [24–27].

**Fig. 5:**
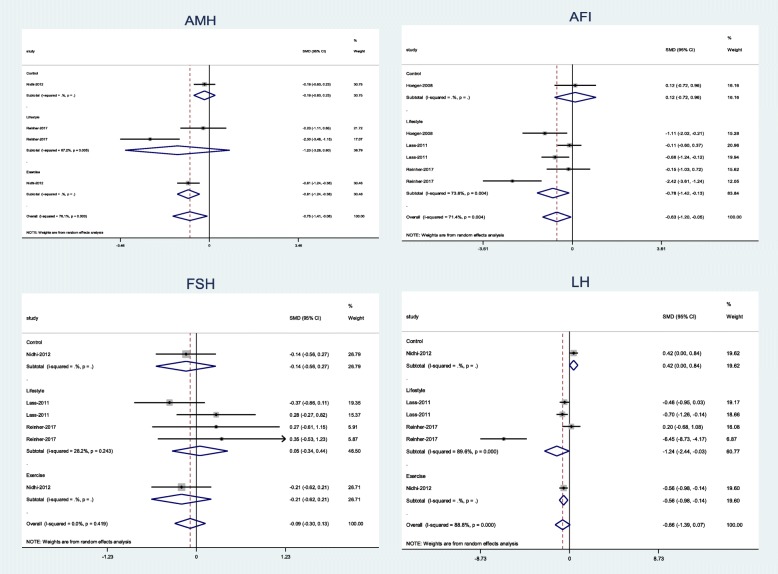
Forest plot of pooled mean difference standardized of hormonal parameters

Meta-analysis results showed that LSM (combination of diet, exercise, and behavior intervention) was significantly associated with a decrease in LH (Pooled SMD = − 0.1.2; 95% CI, − 2.44 to − 0.03), and FAI (Pooled SMD = − 0.78 95% CI, − 0.1.42 to − 0.13). In addition, the exercise intervention significantly decreased LH (Pooled SMD = − 06; 95% CI, − 0.98 to − 0.14) and AMH levels (Pooled SMD = − 0.81; 95% CI, − 0.1.24 to − 0.38) (Fig. 5).

LSM had no significant effects on other hormonal parameters, including FSH, SHBG, TT, FT, BT levels (Figs. 5, 6 in Additional file 1).

### Meta-regression analysis

The results of meta-regression showed differences in the clinical, hormonal, and metabolic parameters between interventions and control groups. Our univariate meta-regression analysis found no significant effects between lifestyle intervention types compared to the control group (Table 2). In addition, the results of meta-regression adjusted for duration of follow-up showed no significant effects of this confounder (Table 3).

**Table 3:** Results of effect of follow-up time in Meta-regression analysis

Outcomes	^a^Meta-regression coefficient
	(***P***-Value)
**BMI**	−.005669 (0.911)
**FG score**	.0198017 (0.878)
**SBP**	−.1612566 (0.245)
**DBP**	.1273615 (0.353)
**Menstrual cycle**	@
**FBS**	.010024 (0.945)
**fasting blood insulin**	.0809897 (0.596)
**HOMA-IR**	@
**TG**	−.0863414 (0.439)
**SHBG**	.0365641 (0.785)
**HDL**	.0754495 (0.403)
**LDL**	.0311606 (0.720)
**FSH**	.0223895 (0.639)
**LH**	−.2349961 (0.564)
**TT**	.0136399 (0.944)
**FT**	@
**BT**	@
**AFI**	.0597252 (0.778)
**AMHr**	@

^a^Meta-regression coefficient showed difference of intervention vs. control

@ insufficient data

### Publication Bias

The results of the Egger test showed no significant publication bias for clinical, metabolic, and hormonal variables.

## Discussion

This study compared the effects of LSM on anthropometric, clinical, and biochemical parameters in adolescent girls with PCOS. In this meta-analysis that included 11 studies involving 412 adolescent girls, we observed that LSM, especially exercise was significantly associated with an improvement in some clinical, metabolic and hormonal findings of PCOS in this population.

In general, the main lifestyle interventions recommended for adolescents with PCOS include exercise, diet, and behavior habit modifications. It has been suggested that lifestyle modification (LSM) can improve menstrual cycle disorders, insulin resistance, and hyperandrogenism through reducing energy intake and weight management [40, 41].

In line with the mentioned mechanisms, the result of our study demonstrated that LSM for 3 to 12 months can significantly decrease the levels of LH and FAI, although we did not observe any changes in other hormonal parameters (FSH, TT, SHBG, and FT, BT, and AMH). We also found that LSM can significantly decline TG levels, whereas it did not affect the levels of other metabolic parameters.

Previous studies on PCOS patients reported that LSM is associated with an increase in spontaneous pregnancy through the restoration of ovulatory cycles [42, 43]. A Cochrane review on 15 studies with 498 participants suggested that LSM can improve body composition in women with PCOS [44], which is similar to our findings. On the contrary, a meta-analysis of 12 RCTs demonstrated that LSM alone for 6 months cannot improve reproductive outcomes (menstrual irregularity, and hirsutism), and metabolic features [45]. This discrepancy might be related to differences in studied populations (adult women vs. adolescents), duration of intervention, and type of lifestyle interventions.

Most included studies in the meta-analysis evaluated the impact of exercise and diet therapy as LSM [24, 25, 29, 34]. There is evidence demonstrating that exercise is associated with significant improvements in ovarian morphology, and ovulatory cycles, mainly through decreasing plasma TNF-α, and increasing plasma IL-4 and IL-10. In addition, it is well-documented that decreased insulin concentration during intensive exercises can improve hirsutism, acne, and menstrual regularity [46].

The present study showed that exercise intervention (yoga) was associated with a significant improvement in clinical manifestations of PCOS, including the FG score, and menstrual cycle irregularity. In addition, exercise significantly diminished serum levels of LH, FAI, and AMH. Moreover, this study showed significant improvement in TG levels after intervention with exercise, whereas there were no changes in the levels of other metabolic parameters with this intervention. Yoga is an alternative exercise activity that might be effective in improving anxiety and mood of adolescence [47], and ovarian morphology in PCOS adult women [48].

Similar to our findings, two other studies showed that weight reduction, through increasing physical exercise alone, can be effective in regulating menstrual cycles [11, 49]. Some studies demonstrated that in addition to physical activity, eating habits and stressful lifestyles have direct effects on the menstrual regularity in adolescent girls [50–52] and adult women [53]. In agreement with our results, a meta-analysis of 27 studies of adult women with PCOS has shown beneficial effects of exercise alone in terms of metabolic factors and anthropometric outcomes [54]. In addition, another meta-analysis of 14 studies involving 617 adult women with PCOS found that exercise improved lipid profiles and decreased waist circumference, systolic blood pressure and fasting insulin, whereas the impact of exercise interventions on reproductive function remained unclear [55].

Evidence suggests that environmental factors such as dietary habits play an important role in the prevention and treatment of PCOS [56]. Indeed, dietary modifications alone, either through qualitative changes or caloric restriction, may improve insulin resistance and hyperandrogenism in PCOS patients [56, 57]. Also, a 5% weight loss through low-calorie diets can improve reproductive system dysfunctions, and fertility in these women [2]. Data for effects of diet therapy alone on clinical and biochemical parameters of PCOS were limited, but general diet therapy (hypocaloric diet with Low Fat: LF or Low-Glycemic-Load: LGL intervention) was associated with a significant decrease in the BMI, and FG score, whereas this intervention had no significant effects on menstrual cycles, and hormonal and metabolic parameters. One meta-analysis of eight studies in PCOS adult women recommended a hypocaloric diet for the reduction of BMI, treatment of PCOS with insulin resistance, prevention of high LDL-C, increasing the levels of FSH and SHBG, and decreasing the level of TG level [58].

In this study, LSM was not significantly associated with changes in glucose metabolism, whereas other meta-analyses reported that LSM in adult PCOS women reduced fasting blood glucose, insulin levels and insulin resistance [15, 59]. We would like to note that intensive lifestyle modification and weight reduction are necessary to reduce circulating insulin and androgen levels [46] and this conflict might be due to the fact that intensive LSM in adolescents might not be a common phenomenon. Indeed, it has been proposed that weight management with LSM interventions may be less effective in adolescent girls with PCOS. This might be related to the hormonal changes of PCOS such as hyperandrogenemia or insulin resistance, contributing to abnormalities in energy homeostasis and dietary intake including gastrointestinal hormone regulation or a modified metabolism because of reduced thermogenesis following meals [60]. Moreover, it was stated that the intensity of hyperinsulinemia and insulin resistance (which has a great effect on the phenotype of PCOS) is further influenced by both genetic factors (such as polymorphism in the insulin gene regulatory region) and environmental factors, especially obesity. Therefore, the efficacy of LSM on insulin resistance might be affected by study populations [61].

In this study, serum levels of lipids showed no significant changes with LSM intervention except in TG level, and the findings were similar to those of other studies in PCOS adults [15, 44, 59, 62]. We observed that LSM was less effective in glucose metabolism in adolescent girls with PCOS.

The present study did not show a significant effect of LSM on the values of SBP and DBP. Similarly, a meta-analysis on adult women with PCOS found no effect of LSM on blood pressure [54], although systematic review studies in hypertension women demonstrated that LSM had significant effects on reducing blood pressure in PCOS [63] and non-PCOS women [64, 65]. This disagreement could be due to differences in the type and duration of interventions and populations studied [63].

It must be noted that there is no strong evidence on the type, duration, and intensity of these interventions to improve clinical and biochemical manifestations of this syndrome in adolescent girls. However, some guidelines have recommended that physical activity of longer duration, higher frequency, and intensity result in better maintenance of health. Importantly, moderate to vigorous daily physical activity for at least 60 min is related to better physical and psychosocial health in children and adolescents [66]. Although the low-glycemic diet in PCOS is recommended [46], international guidelines have demonstrated that there is limited evidence as to which specific energy equivalent diet type is better [67]. In addition, based on the international guidelines, a duration of 6 to 12 months is the minimum time for the effects of LSM to show. Moreover, guidelines recommend synchronous use of diet, exercise and psychology changes [67].

The main strength of this review was its novelty as the first meta-analysis assessing the effect of lifestyle on PCOS adolescents. In addition, in this study, potential confounders, including age, and diagnostic criteria were adjusted and subgroup analysis was performed based on the type of lifestyle intervention.

Our study had some limitations that should be considered in order to interpret the results. The main limitation of this study was the small number of publications assessing the impact of lifestyle modifications in adolescent girls with PCOS, and hence; we were unable to conduct subgroup analysis based on all interventions and outcomes of interest. Also, we could not assess type and intensity of the interventions for exercise and diet therapy due to insufficient data. Although, because of insufficient data, we could not conduct a subgroup analysis based on the follow-up duration, results of our meta-regression showed no significant effect of this confounder. In most studies included in our analysis, LH levels were evaluated in the follicular phase of the menstrual cycle except in participants with oligo or amenorrhea. Due to this problem, lower LH levels after LSM in our results might not be extended to all PCOS adolescents. It should be kept in mind that during adolescence, height is not stabilized, which could affect BMI measurements. There was significant heterogeneity in most outcomes where random effect analysis was done to deal with this heterogeneity. These heterogeneities might be related to clinical heterogeneity, which can be due to variability in PCOS diagnostic criteria and laboratory tests, and study population (e.g., age, BMI, ethnicity or race).

## Conclusion

Our analysis suggests that, although lifestyle modification through reduced calorie intake and regular physical activity can improve clinical, metabolic, and hormonal parameters in adolescent girls with PCOS, further well-designed studies are still required to elucidate and confirm these findings.

## Supplementary information

**Additional file 1.**

## Data Availability

The datasets used and/or analyzed during the current study are available from the corresponding author on reasonable request.

## Abbreviations

PCOS: Polycystic Ovarian Syndrome
BMI: Body Mass Index
FSH: Follicle-Stimulating Hormone
LH: Luteinizing Hormone
FBS: Fasting Blood Sugar
HOMA: Homeostatic Model Assessment
FBI: Fasting Blood Index
TG: Triglyceride
LDL: Low-density lipoprotein
HDL: High-density lipoprotein
SHBG: Sex Hormone Binding Globulin
TT: Total Testosterone
FT: Free Testosterone
BT: Bioavailable Testosterone
FAI: Free Androgen Index
AMH: Anti-Müllerian Hormone
BP: Blood Pressure
FG: Ferriman-Gallwey
